# Impact of skilled nursing facility discharge on inpatient oncology quality outcomes

**DOI:** 10.1093/jncics/pkaf055

**Published:** 2025-05-23

**Authors:** Bonnie E Gould Rothberg, Jensa C Morris

**Affiliations:** Department of Medicine, University of Miami Miller School of Medicine, Miami, FL, United States; Smilow Hospitalist Service, Yale-New Haven Hospital, New Haven, CT, United States; Division of General Internal Medicine, Department of Internal Medicine, Yale School of Medicine, New Haven, CT, United States

## Abstract

**Background:**

Hospitalist comanagement of inpatient oncology patients can improve length of stay (LOS), discharge time, and readmission rates. Identifying additional clinical factors affecting LOS and readmissions will guide further oncology hospitalist practice improvement.

**Methods:**

Hospitalizations on the Smilow Cancer Hospital medical oncology service with discharge to home under self-care (*n* = 622), home with services (*n* = 462), or skilled nursing facility (*n* = 152) from July 1, 2021, to July 31, 2022, were included. Outcomes included LOS, time of discharge, and 30-day readmission rate. Multivariable mixed linear (LOS, time of discharge) or Poisson (30-day readmission rates) models were adjusted for demographics, cancer type, severity of illness index, house staff team, and fiscal quarter and included a random intercept for patient. Analyses were 2-sided with a priori statistical significance of less than 0.05.

**Results:**

Patients discharged to a skilled nursing facility had a longer LOS (mean = 8.25 days, 95% confidence interval [CI] = 7.13 to 9.55 days) compared with patients discharged to home under self-care (mean = 3.04 days, 95% CI = 2.76 to 3.36 days) or with services (mean = 4.48 days, 95% CI = 4.03 to 4.97 days; *P *< .0001). Thirty-day readmission rates for patients discharged to a skilled nursing facility (43.99%) were 10 percentage points higher than those discharged home either under self-care (32.86%) or with services (33.48%; *P *= .14). These differences persisted in patients regardless of severity of illness index. Of the 152 patients discharged to a skilled nursing facility, 31 (20.3%) were readmitted specifically back to the medical oncology service within 60 days with 16 (51.6%) cycling back to a skilled nursing facility, which resulted in 11 second readmissions.

**Conclusion:**

For oncology patients requiring discharge to a skilled nursing facility, mindful and upfront discharge planning may improve care quality and efficiency.

## Introduction

Hospital overcrowding is pervasive. High inpatient occupancy rates are associated with delays in transfers out of the emergency department (ED),^1^ postanesthesia care units,^2^ and intensive care units.^3^ This patient gridlock delays essential care and increases mortality, cost, and inpatient hospital days.^4-6^ The scarcity of inpatient beds is magnified for cancer patients who require specialized treatments best delivered on dedicated oncology units.^7-9^ Over the past decade, many cancer centers have incorporated oncology hospitalists as the primary attendings for cancer inpatients.^10-12^ The positive impact of hospitalists on process and efficiency metrics including length of stay (LOS) and overall cost is well established.^13^^,^^14^ We^12^ as well as others^10^^,^^11^^,^^15^^,^^16^ have shown that adoption of an oncology hospitalist program can meaningfully reduce LOS and ameliorate patient flow without impacting 30-day readmission rates. We further demonstrated that oncology hospitalists reduce LOS through improved hospice use and decreased time to inpatient hospice referral.^17^ Despite improved care efficiency with oncology hospitalists at our institution, patients requiring specialized cancer care continue to board in the ED and overflow to general medical services because of limited inpatient bed capacity. With the goal of driving further improvements in inpatient care, we conducted a cross-sectional analysis to characterize the impact of discharge disposition on quality and efficiency measures with a focus on the impact of discharge to postacute care.

## Methods

### Setting

The setting reflected patients hospitalized on the inpatient medical oncology service within Smilow Cancer Hospital (SCH) at the Yale Cancer Center. All acutely ill oncology patients, largely with advanced-stage disease, are preferentially admitted to the SCH inpatient solid tumor service when beds are available. When SCH is at capacity, patients are either temporarily held in the ED or redirected for admission to the general medical service. The SCH inpatient solid tumor service comprises 2 teams, each with house staff who care for 9 patients each; advanced practice providers manage the remaining patients. During this study period, 1 team was led by an oncologist, and the other was led by a hospitalist. All inpatient discharges from the solid tumor service to home or skilled nursing facility from July 1, 2021, to July 31, 2022, were included; contemporaneous hospitalizations billed as observation (*n *= 212) or hospice (*n *= 36) or discharged as expired (*n* = 34) or against medical advice (*n* = 6) were excluded. Patients discharged to home hospice (*n* = 63) or inpatient hospice (*n* = 132) were excluded because the results were reported elsewhere.^17^ The Yale University institutional review board considered this study to be quality improvement and required no further action.

### Patient Characteristics

To flag patients with multiple admissions, a unique patient identifier derived from the medical record number (PTID) was assigned to each patient. Discharge disposition was extracted from the discharge summary and defined as home under self-care, home with services, or discharge to skilled nursing facility for short-term rehabilitation or long-term care. Demographic variables were extracted from the medical record. Age was categorized as younger than 55 years, 55-69 years, and 70 years and older. Sex was categorized as male or female. Race and ethnicity were categorized as non-Hispanic White, Black, Hispanic, and Asian; patients with missing (*n* = 23) or other (*n* = 1) values were excluded. Health insurance type was extracted from the patient’s billing record and classified as Blue Cross Blue Shield or Commercial, Managed Care, Medicaid, Medicare, and Medicare Managed Care; individuals with self-pay (*n* = 8) were excluded. Cancer type corresponded to the malignancy coded along the patient’s most recent line of chemotherapy. Chart review was conducted by a single investigator (B.E.G.R.) to resolve ambiguous entries and was subsequently grouped into systems-based categories. Cancer types with fewer than 10 patients were compiled as “other.” The severity of illness index was autocalculated within the electronic health record based on the diagnosis-related group associated with the index admission primary diagnoses combined with coded comorbidities and scored on a scale of 1-4.^18^ Team attending of record was defined as “traditional” for the oncologist-led team and “hospitalist” for the hospitalist-led team. Fiscal quarter was derived from the date stamp of the discharge date. The resultant dataset was complete across all independent covariates.

### Outcome Metrics

LOS, discharge time, and 30-day readmission rates are reported. LOS is calculated as the number of hours elapsed between the time stamps for admission and discharge and is reported in days. Discharge time, reported in military time, is derived from the time stamp when the unit secretary removed the patient from the inpatient census and converted to the total minutes elapsed following midnight of the calendar day of discharge for statistical modeling. Readmission date is extracted through query of the Yale-New Haven Health System database for the first subsequent inpatient admission following the discharge date stamp. First readmissions occurring up to 365 days following the index discharge were captured with a flag generated if this readmission occurred within 30 days.

### Skilled nursing facility readmission outcomes

An exploratory analysis of readmissions from skilled nursing facilities was conducted. All readmissions from skilled nursing facilities where the readmission was to the inpatient solid tumor service occurring within 60 days of the index discharge and during our study time frame were included. The number of days until readmission, the LOS of the readmission, the discharge disposition, and whether this rehospitalization resulted in a subsequent second readmission were captured.

### Data analysis

The distribution of sociodemographic and clinical covariates according to discharge disposition were tabulated and bivariate associations were conducted using the likelihood ratio χ^2^ test. Analyses with LOS and time of discharge were conducted using mixed linear regression. LOS was modeled following natural log transformation with point estimates and 95% confidence intervals (CIs) then reflecting the natural exponentiation of the fitted regression values. Time of discharge, being normally distributed, did not require transformation prior to linear regression analysis. Residual analyses confirming underlying assumptions were verified for each model. Thirty-day readmission rates were conducted by mixed Poisson regression. All mixed models included a first-order random intercept for patient to account for the subset of patients with multiple admissions during the study interval and employed a variance components covariance matrix structure with its convergence verified. Multivariable models were built using forward selection to include the variables for age, sex, race and ethnicity, insurance type, cancer type, discharge disposition, severity of illness index, team attending, and fiscal quarter. In all cases, variables were entered in ascending order of univariate *P* value until either all variables were included in the final model or until the model global maximum likelihood estimate did not converge. In the latter case, the last stable model was considered as the final model. All statistical analyses were conducted in SAS version 9.4 (SAS Institute, Cary, NC, USA). All models are 2-sided, and statistical significance was defined a priori as a *P* value less than .05.

## Results

Altogether, 1236 eligible hospitalizations were distributed among 848 unique individuals with 588 participants having 1 hospitalization, 231 with 2 or 3 hospitalizations, and 29 with 4 or more eligible hospitalizations. Of the patients, 152 (12.3%) were discharged to a skilled nursing facility, including 148 anticipating short-term rehabilitation and 4 to long-term acute care. Statistically significant associations with discharge disposition were noted for age, health insurance payer, anatomic site of the cancer primary, and severity of illness index (all *P*s < .0001; Table 1).

**Table 1.** Demographic and clinical characteristics of hospitalizations

Covariate	Home, self-care, No. (%) (*n* = 622)	Home, with services, No. (%) (*n* = 462)	Skilled nursing facility, No. (%) (*n* = 152)	*P*
Severity of illness index				
1 or 2	286 (46.0)^a^	128 (27.8)	26 (17.1)	<.0001
3 or 4	336 (54.0)	333 (72.2)	126 (82.9)	
Age, y				
Younger than 55	129 (20.7)	72 (15.6)	23 (15.1)	<.0001
55-69	288 (46.3)	181 (39.2)	51 (33.6)	
70 and older	205 (33.0)	209 (45.2)	78 (51.3)	
Sex				
Male	314 (50.5)	207 (44.8)	76 (50.0)	.16
Female	308 (49.5)	255 (55.2)	76 (50.0)	
Race and ethnicity				
Asian	15 (2.4)	13 (2.8)	1 (0.7)	.10
Black	68 (10.9)	74 (16.0)	21 (13.8)	
Hispanic	54 (8.7)	41 (8.9)	10 (6.6)	
Non-Hispanic White	485 (78.0)	334 (72.3)	120 (79.0)	
Health insurance payer				
Blue Cross Blue Shield/Commercial	126 (20.3)	59 (12.8)	13 (8.6)	<.0001
Managed Care	109 (17.5)	46 (10.0)	6 (4.0)	
Medicaid	87 (14.0)	77 (16.7)	27 (17.8)	
Medicare	185 (29.7)	129 (27.9)	63 (41.5)	
Medicare Managed Care	115 (18.5)	151 (32.7)	43 (28.3)	
Anatomic site of the primary cancer				
Thoracic	88 (14.2)	109 (23.6)	36 (23.7)	<.0001
Upper gastrointestinal	192 (30.9)	117 (25.3)	22 (14.5)	
Lower gastrointestinal	84 (13.5)	34 (7.4)	7 (4.6)	
Breast	80 (12.9)	56 (12.1)	25 (16.5)	
Genitourinary	73 (11.7)	72 (15.6)	29 (19.1)	
Head and neck	37 (6.0)	27 (5.8)	18 (11.8)	
Melanoma or sarcoma	49 (7.9)	35 (7.6)	10 (6.6)	
Other^b^	19 (3.1)	12 (2.6)	5 (3.3)	
Covering team				
Academic team 1, traditional	308 (49.5)	238 (51.5)	78 (51.3)	.79
Academic team 2, hospitalist	314 (50.5)	224 (48.5)	74 (48.7)	
Fiscal quarter				
January 1-March 31	133 (21.4)	114 (24.7)	28 (18.4)	.48
April 1-June 30	146 (23.5)	103 (22.3)	33 (21.7)	
July 1-September 30	180 (28.9)	134 (29.0)	55 (36.2)	
October 1-December 31	168 (26.2)	111 (24.0)	36 (23.7)	

a Numbers may not sum to total because of missing; percentages may not sum to 100% because of rounding.

b Other includes all cancer types with fewer than 10 cases with adrenal, astrocytoma and glioblastomas, gynecologic, Merkel cell and nonmelanoma skin cancers, thyroid, and cancers of unknown primary represented.

The median LOS was 3.87 days (interquartile range [IQR] = 2.20-7.05 days). In multivariate analysis, patients with severity of illness index of 3 or 4 (high severity of illness index; mean LOS = 6.02 days, 95% CI = 5.45 to 6.65 days) stayed nearly 1.5 days longer than patients with severity of illness index of 1 or 2 (low severity of illness index; mean LOS = 3.87 days, 95% CI = 3.47 to 4.33 days; *P* < .0001). Similarly, compared with patients discharged home under self-care (mean LOS = 3.04, 95% CI = 2.76 to 3.36 days), patients discharged to skilled nursing facility had a 5-day-longer hospitalization (mean LOS = 8.25 days, 95% CI = 7.13 to 9.55 days; *P *< .0001) (Table 2). When discharge disposition and severity of illness index were modeled jointly, patients with low severity of illness index discharged home under self-care had a mean LOS of 2.30 days (95% CI = 1.98 to 2.70 days), whereas similarly low severity of illness index patients discharged to a skilled nursing facility had a mean LOS of 6.08 days (95% CI = 4.46 to 8.28 days; *P *< .0001). Accordingly, patients with high severity of illness index discharged home under self-care had a mean LOS of 3.96 days (95% CI = 3.47 to 4.52 days), whereas similarly high severity of illness index patients discharged to a skilled nursing facility had a mean LOS of 10.55 days (95% CI = 8.91 to 12.49 days; *P *< .0001) (Table 3).

**Table 2.** Quality outcomes within an inpatient medical oncology unit; July 1, 2021-July 31, 2022

Outcome measure	Univariate (95% CI)	*P*	Multivariate (95% CI)	*P*
Length of stay, d^a^				
Severity of illness index				
1 or 2, low	2.85 (2.64 to 3.08)	<.0001	3.87 (3.47 to 4.33)	<.0001
3 or 4, high	5.04 (4.75 to 5.34)		6.02 (5.45 to 6.65)	
Discharge disposition				
Home, self-care	2.98 (2.81 to 3.17)	<.0001	3.04 (2.76 to 3.36)	<.0001
Home, with services	4.80 (4.47 to 5.15)		4.48 (4.03 to 4.97)	
Skilled nursing facility	9.27 (8.20 to 10.47)		8.25 (7.13 to 9.55)	
Discharge time, h:min^b^				
Severity of illness index				
1 or 2, low	15:01 (14:46 to 15:16)	<.0001	15:31 (15:07 to 16:06)	.01
3 or 4, high	15:38 (15:27 to 15:50)		15:57 (15:35 to 16:19)	
Discharge disposition				
Home, self-care	14:57 (14:44 to 15:09)	<.0001	15:08 (14:45 to 15:30)	<.0001
Home, with services	15:44 (15:30 to 15:59)		15:47 (15:23 to 16:10)	
Skilled nursing facility	16:23 (15:58 to 16:48)		16:18 (15:45 to 16:51)	
30-day readmissions, %^a^				
Severity of illness index				
1 or 2, low	30.00% (25.28% to 35.60%)	.054	33.01% (25.54% to 42.65%)	.07
3 or 4, high	36.73% (32.74% to 41.21%)		40.24% (32.31% to 50.11%)	
Discharge disposition				
Home, self-care	32.96% (28.73% to 37.81%)	.22	32.86% (26.16% to 41.28%)	.14
Home, with services	33.77% (28.84% to 39.52%)		33.48% (26.34% to 42.56%)	
Skilled nursing facility	42.11% (32.93% to 53.84%)		43.99% (31.97% to 60.53%)	

Abbreviation: CI = confidence interval.

a Adjusted for age, sex, race and ethnicity, health insurance payer, anatomic location of the cancer primary, severity of illness index, discharge disposition, attending team of record, and fiscal quarter.

b Adjusted for age, race and ethnicity, health insurance payer, anatomic location of the cancer primary, severity of illness index, discharge disposition, attending team of record, and fiscal quarter (full model that also included health insurance payer was unstable).

**Table 3.** Quality outcomes for the joint distribution of Severity of Illness Index and discharge disposition

Outcome measure	Univariate estimate (95% CI)	*P*	Multivariable estimate^a^ (95% CI)	*P*
Length of stay, d				
Low severity of illness index (1 or 2)				
Home, self-care	2.42 (2.21 to 2.64)	<.0001	2.30 (1.98 to 2.70)	<.0001
Home, with services	3.49 (3.07 to 3.97)		3.31 (2.76 to 3.97)	
Skilled nursing facility	6.38 (4.82 to 8.43)		6.08 (4.46 to 8.25)	
High severity of illness index (3 or 4)				
Home, self-care	3.62 (3.35 to 3.93)	<.0001	3.96 (3.47 to 4.52)	<.0001
Home, with services	5.41 (4.99 to 5.86)		5.77 (5.07 to 6.55)	
Skilled nursing facility	9.89 (8.69 to 11.26)		10.55 (8.91 to 12.49)	
Time of discharge, h: min				
Low severity of illness index (1 or 2)				
Home, self-care	14:37 (14:19 to 14:56)	<.0001	14:43 (14:11 to 15:15)	<.0001
Home, with services	15:55 (15:28 to 16:22)		16:03 (15:25 to 16:40)	
Skilled nursing facility	15:40 (14:40 to 16:39)		15:49 (14:45 to 16:53)	
High severity of illness index (3 or 4)				
Home, self-care	15:18 (15:01 to 15:34)	<.0001	15:40 (15:11 to 16:09)	.001
Home, with services	15:43 (15:27 to 16:00)		16:01 (15:33 to 16:29)	
Skilled nursing facility	16:31 (16:05 to 16:58)		16:46 (16:09 to 17:23)	
30-day readmission rate, %				
Low severity of illness index (1 or 2)				
Home, self-care	29.02% (23.32% to 27.69%)	.84	22.42% (14.03% to 35.82%)	.45
Home, with services	31.25% (22.81% to 42.82%)		26.22% (15.51% to 44.30%)	
Skilled nursing facility	34.61% (17.82% to 67.23%)		34.79% (15.75% to 76.84%)	
High severity of illness index (3 or 4)				
Home, self-care	36.31% (30.37% to 43.41%)	.35	38.37% (28.85% to 51.03%)	.23
Home, with services	34.53% (28.73% to 41.51%)		36.12% (27.45% to 47.54%)	
Skilled nursing facility	43.65% (33.45% to 56.96%)		48.19% (33.92% to 68.43%)	

Abbreviation: CI = confidence interval.

a Multivariable model adjusted for age, sex, race and ethnicity, health insurance payer, anatomic location of the cancer primary, attending team of record, and fiscal quarter.

The median discharge time was 15:40 (IQR = 13:34-17:21). Discharge time varied statistically significantly according to both severity of illness index and discharge disposition. Mean discharge time was latest for patients discharged to a skilled nursing facility (mean = 16:18, 95% CI = 15:45 to 16:51; *P *< .0001), more than 1 hour longer than patients discharged to home under self-care (mean = 15:08, 95% CI = 14:45 to 15:30) (Table 2). The difference in discharge times is most notable in patients with low severity of illness index, whereby patients discharged home under self-care (mean discharge time = 14:43, 95% CI = 14:11 to 15:15) were discharged statistically significantly earlier than those requiring coordination of home services or postacute care (*P *< .0001). Among high severity of illness index patients, those being discharged to a skilled nursing facility stayed latest (*P *< .0001) (Table 3).

The 30-day readmission rate to all locations within the greater Yale-New Haven Health System for all discharges was 34.39%. Overall, patients discharged to a skilled nursing facility were most likely to be readmitted, although this result did not achieve statistical significance (43.99%, 95% CI = 31.97% to 60.53%; *P *= .14). When stratified by severity of illness index, although not statistically significant because of the wide confidence intervals surrounding patients discharged to a skilled nursing facility, the readmission rate point estimates for low- and high-acuity patients discharged to a skilled nursing facility ranged between 8 and 10 percentage points higher than patients discharged home with services (Table 3).

To better understand the outcomes of patients readmitted from a skilled nursing facility, we conducted an exploratory analysis of the subset of patients specifically readmitted to the Smilow Medical Oncology service within 60 days of their index discharge during the study interval (Table 4). Within the initial cohort, 152 patients were discharged to a skilled nursing facility. Of these, 31 (20.4%) were readmitted to the SCH Medical Oncology service within 60 days, including 24 patients readmitted within 30 days. Of the 31 patients, 16 (51.6%) were subsequently discharged back to a skilled nursing facility of which 11 (68.8%) were readmitted again to SCH. All but 2 of these 11 second readmissions occurred within 30 days. By contrast, 9 (29.0%) of 31 patients readmitted after initial skilled nursing facility discharge were discharged to either home hospice or inpatient hospice. None of these patients were readmitted to SCH. One individual, readmitted from a skilled nursing facility 43 days after discharge, expired in Smilow on hospital day 1.

**Table 4.** Outcomes following a readmission to the Smilow Cancer Hospital from SNF within 60 days of discharge from the index admission (*n* = 31)

Patient	Age, y	Severity of illness index at index admission	Length of stay; index admission, days	Discharge disposition: index admission	Days to first readmission	Length of stay; first readmission, days	Discharge disposition; first readmission	Any subsequent readmission	Days to subsequent readmission
639	54	3	9	SNF	1	6	SNF	No	
637	52	3	48	SNF	2	5	SNF	No	
37	75	3	5	SNF	10	14	SNF	No	
930	69	3	2	SNF	18	5	SNF	No	
68	80	4	15	SNF	37	12	SNF	No	
917	60	2	15	SNF	2	6	SNF	Yes	12
615	51	3	10	SNF	5	10	SNF	Yes	30
839	76	4	6	SNF	6	2	SNF	Yes	17
190	71	3	36	SNF	6	7	SNF	Yes	14
892	69	3	8	SNF	9	4	SNF	Yes	7
633	59	4	25	SNF	11	20	SNF	Yes	37
1060	52	4	2	SNF	12	4	SNF	Yes	7
786	78	4	14	SNF	20	3	SNF	Yes	4
653	49	4	10	SNF	29	5	SNF	Yes	14
423	66	4	5	SNF	39	13	SNF	Yes	56
852	80	4	5	SNF	48	2	SNF	Yes	29
395	69	3	19	SNF	9	5	Hospice, home	No	
771	81	3	9	SNF	28	6	Hospice, home	No	
235	74	3	8	SNF	2	9	Hospice, inpatient	No	
723	70	3	9	SNF	8	8	Hospice, inpatient	No	
401	61	2	13	SNF	16	2	Hospice, inpatient	No	
486	61	3	5	SNF	30	6	Hospice, inpatient	No	
17	64	3	7	SNF	35	19	Hospice, inpatient	No	
825	73	4	14	SNF	50	5	Hospice, inpatient	No	
147	75	3	13	SNF	59	4	Hospice, inpatient	No	
22	71	4	11	SNF	43	1	Expired	—	
659	57	3	13	SNF	8	23	Home with services	No	
273	73	4	14	SNF	11	10	Home with services	No	
553	69	3	5	SNF	24	2	Home with services	No	
136	67	3	22	SNF	2	18	Home with services	Yes	8
773	65	3	15	SNF	19	3	Home with services	Yes	13

Abbreviation: SNF = skilled nursing facility.

## Discussion

Provision of high-quality, high-efficiency inpatient oncology care is a priority. Care delays due to reduced bed access have a detrimental effect on morbidity and mortality,^5^^,^^19^ patient satisfaction,^20^ and hospital finances.^21^ High demand for inpatient beds has mandated that hospitals focus on metrics of care efficiency and quality such as LOS, time of discharge, and readmission rates. Understanding clinical and sociodemographic factors that impact inpatient bed use will inform future priorities for quality improvement initiatives on an inpatient medical oncology service.

The outcomes we report here resemble nationally reported data for acutely ill oncology inpatients. In the United States, mean LOS for oncology inpatients ranges from 4 to 9 days,^22-26^ and readmission rates of oncology patients are reported as 16%-35%.^27-30^ Mean time of discharge is not well reported in the oncology literature, however, rate of discharge before noon has been reported as 4%-22%.^31-33^ Here, we report a median LOS of 3.87 days, a median discharge time of 15:40, and a readmission rate of 34.39%.

Discharge disposition, which incorporates the multistep care coordination required for discharge with home services or to a facility, and severity of illness index, a metric that combines chief complaint acuity with preexisting comorbidities, were independently associated with LOS. Low-acuity patients discharged to a skilled nursing facility had a LOS of more than 2 days longer than similarly low acuity patients discharged home with services. Securing either home-based services or postacute care placement requires effective, interdisciplinary care coordination early during the hospitalization.^34^ Additional barriers for discharge to a skilled nursing facility, however, are awaiting bed availability and securing postacute insurance authorization. This process requires more than 48 hours^35^ and frequently extends beyond the time a patient has achieved medical stability.^36^ This delay can account for a substantial portion of the longer LOS observed among patients with a low severity of illness index discharging to a skilled nursing facility as compared with similar patients discharging home with self-care or home with services.

The association between discharge to a skilled nursing facility and elevated readmission rates is well established in general medicine patients.^37^^,^^38^ Although the data are less robust in cancer populations, studies have similarly demonstrated increased oncology readmissions with a skilled nursing facility discharge.^30^^,^^39^

Discharge to a skilled nursing facility requires that patients meet specific criteria including the requirement for daily physical therapy sessions and/or daily administration of intravenous medications that cannot be completed by the patient’s caregivers or a visiting nurse. In cases of the former, these are usually triggered by physical deconditioning from the index hospitalization.^40^ However, given the substantially improved quality outcomes for patients discharged home with services compared with those discharged to a skilled nursing facility, encouraging the inpatient team to explore barriers to discharge home with the patient and their caregivers may identify additional patients for whom a discharge home can be a safe option. We posit that the subset of patients whose severity of illness index score was low on admission may be the best candidates who, while meeting criteria for discharge to a skilled nursing facility, can safely discharge to home with the appropriate in-home supports.

Reconsidering whether a skilled nursing facility discharge in hospitalized oncology patients with the highest severity of illness index is the most appropriate discharge disposition is equally important but more challenging. Here, there are multiple conflicting dilemmas to be considered. First, for patients who still wish to receive active therapy, because chemotherapy is considered an outpatient treatment by Medicare and most private payors, treatment cannot be administered in a skilled nursing facility as it is considered an inpatient venue. This places the patient at risk for progression of disease while undergoing rehabilitative care. For frail patients with substantial disease burden, this delay in care might be catastrophic.^39^ Next, there is a subset of patients for whom the new level of deconditioning represents an irreversible deterioration that signals the onset of end of life. Given the established overall survival of less than 50% at 6 months for patients with solid tumors and an unplanned hospital admission,^28^ this index hospitalization presents an opportunity to reevaluate goals of care. Educating patients on their expected prognosis, future treatment options, and expected impact on quality of life is critical at this juncture. Unfortunately, not all high-acuity patients with a limited prognosis who have opted for symptom-based care will qualify for inpatient hospice. The strict criteria for general inpatient hospice care have resulted in most hospice care now being delivered outpatient.^41^ The cost and family time required to provide supportive care at home^42^ is prohibitive for many. When patients do not qualify for general inpatient hospice and home hospice is not an option because of resources, skilled nursing facility discharge is often the default. Of the 31 patients in our exploratory analysis, 9 readmitted from a skilled nursing facility within 60 days were ultimately discharged to hospice. It is important to note, however, that more than half of these patients were readmitted from a skilled nursing facility 25 or more days after discharge to make it plausible that, although they might have been too frail for a safe discharge home, they did not yet qualify for inpatient hospice. Because discharging patients to a skilled nursing facility at the end of life is neither patient centered nor an efficient use of resources, one important policy improvement will be to broaden the accessibility of hospice care for end-of-life cancer patients.

Skilled nursing facility discharge rates vary widely by geographic region, insurance payor, patient race, community income, and type of discharging hospital (academic vs community).^43^ This variation is independent of patient acuity or medical complexity. For example, discharge rates to a skilled nursing facility are highest in the Northeast United States with Connecticut discharging patients to skilled nursing facilities at rates 27% higher than the national norm.^44^ Discharge disposition is therefore a variable over which the inpatient team may have substantial influence. Changing inpatient culture regarding skilled nursing facility discharge will require reeducation of inpatient clinical staff, patients, and families. Educational efforts that focus on discharge outcomes for oncology patients discharged to a skilled nursing facility^39^ may be considered to shift clinician attitudes to favor home discharge. In cases in which skilled nursing facility discharge cannot be avoided, higher-level administrative efforts addressing the extrinsic barriers to skilled nursing facility discharge, such as delays in skilled nursing facility availability, insurance authorization and discharge transportation, are essential next steps.

The discharge disposition to LOS relationship can also be viewed from the reverse perspective whereby patients with a longer LOS are more likely to require a skilled nursing facility following discharge. For example, high-acuity, medically complex patients would be expected to stay longer and are more likely to need a higher level of care at discharge. This high-acuity patient population is unlikely to have a clinically significant LOS reduction simply with improvement in our discharge care processes. This was seen in a cohort of colorectal cancer patients.^45^ In this population, the authors showed that when adjusted for severity of illness index, LOS of 10 days or more was an independent risk factor for skilled nursing facility discharge. The correlation of LOS and skilled nursing facility discharge, independent of severity of illness index, suggests that early and aggressive mobility programs^46^ may be important to reduce skilled nursing facility discharges in the longer LOS cohort.

As it has been shown that hours spent boarding in the ED can pose a substantial safety issue,^5^ improving inpatient flow through promoting earlier inpatient discharge times can provide some ED decompression.^47^ Data reported here can help guide our early discharge efforts. We showed a 1- to 2-hour difference between the discharge time of our low-acuity patients discharged home compared with other patient groups. Patients requiring home services or a skilled nursing facility had later median discharge times. In our experience, this is related to the time-consuming and complex discharge care coordination required. As we manage our workflows and patient expectations to optimize efficiency, an earlier-in-the-day discharge target seems most suitable for low-acuity patients discharging home with or without services.

Strengths of our study include a large, curated, complete dataset extending over a full calendar year supplemented with robust statistical methods. Limitations include focus on a single cancer center, which compromises generalizability, and the lack of data on social determinants of health, which have been previously associated with LOS and 30-day readmissions.^48^ Furthermore, we excluded self-pay patients as their small number precluded statistical modeling. Our inability to account for cultural sensitivities and preferences for discharge disposition may have impacted the outcomes presented here.

Detailed analysis of the SCH inpatient oncology population yields important data for quality improvement work to reduce LOS, time of discharge, and readmission rates. Care coordination to optimize selection of patients most appropriate for discharge to a skilled nursing facility rather than discharge to home with services or to hospice was identified as a clinically relevant opportunity for improved care efficiency. Future studies should assess opportunities to better match discharge disposition to a patient’s functional status, prognosis, and goals for future care.

## Data Availability

A deidentified version of the study data can be made available to individuals following written request to the corresponding author.
